# CONSTRUCT: an algorithmic tool for identifying functional or structurally important regions in protein tertiary structure

**DOI:** 10.1093/bioinformatics/btaf166

**Published:** 2025-04-12

**Authors:** Lucas Chivot, Noé Mathieux, Anna Cosson, Antoine Bridier-Nahmias, Loïc Favennec, Jean-Christophe Gelly, Jérôme Clain, Romain Coppée

**Affiliations:** Université de Rouen Normandie, Laboratoire de Parasitologie-Mycologie, ESCAPE, F-76000 Rouen, France; Université de Rouen Normandie, Laboratoire de Parasitologie-Mycologie, ESCAPE, F-76000 Rouen, France; Université de Rouen Normandie, Laboratoire de Parasitologie-Mycologie, ESCAPE, F-76000 Rouen, France; Université Paris Cité et Sorbonne Paris Nord, Inserm, IAME, F-75018 Paris, France; Université de Rouen Normandie, Laboratoire de Parasitologie-Mycologie, ESCAPE, F-76000 Rouen, France; Université Paris Cité et Université des Antilles et Université de la Réunion, Inserm, BIGR, F-75015 Paris, France; Université Paris Cité, IRD, Inserm, MERIT, F-75006 Paris, France; Université de Rouen Normandie, Laboratoire de Parasitologie-Mycologie, ESCAPE, F-76000 Rouen, France

## Abstract

**Motivation:**

Evolutionary rates in protein-coding genes vary widely, reflecting functional and/or structural constraints. Essential or highly expressed proteins tend to evolve more slowly, and within a protein, different amino acid sites experience distinct selective pressures. Accurately modeling this variation is critical for identifying functional and/or structurally important amino acid sites. Standard methods assume independent substitution rates across sites, and the most conserved ones are widely distributed in protein tertiary structure. This is biologically unrealistic, as functional sites tend to cluster in 3D space.

**Results:**

Here, we developed CONSTRUCT, an improved strategy for detecting functional and structurally important regions in protein tertiary structure. Given a set of orthologous sequences, CONSTRUCT first estimates site-specific substitution rates using the Rate4site model. These rates are then weighted by the rates of neighboring amino acid sites within an optimally defined window size, determined by the strongest spatial correlation. To refine clustering detection, CONSTRUCT can analyze either Cα atoms or the center of mass of amino acid sites, accounting for side chain orientation. Extensive simulations and validation on 14 functionally characterized proteins of diverse sizes, interspecies conservation levels, and taxonomic origins demonstrated the robustness of CONSTRUCT. The results highlight CONSTRUCT as a powerful tool for guiding site-directed mutagenesis experiments aimed at elucidating protein function.

**Availability and implementation:**

The CONSTRUCT program and documentation are freely available at https://github.com/Rcoppee/CONSTRUCT.

## 1 Introduction

In the realm of protein-coding genes, evolutionary rates vary widely within a species. Genes encoding essential or abundantly expressed proteins evolve more slowly (Kimura and Ohta 1974, Zhang and Yang 2015). Within the same protein, amino acid sites also exhibit different rates of evolution. Some of this variation in evolutionary rates arises from selection, either positive such as adaptation to environmental changes or negative (*id est* purifying selection) to maintain critical protein activities (Echave *et al.* 2016). Accurately modeling this variation is critical in evolutionary studies (Yang 1996), particularly for identifying amino acid sites within proteins that are functional or structurally important (Capra and Singh 2007). For that, integrating structural information into phylogenetic models enhances the detection of evolutionary constraints and improves the accuracy of functional site prediction (Echave 2019, Ferreiro *et al.* 2024).

Indeed, research has shown that site-specific evolutionary rates are shaped by both structural and functional constraints (Franzosa and Xia 2009, Nevin Gerek *et al.* 2013, Yeh *et al.* 2014). In particular, less critical parts of a molecule tend to evolve more rapidly than essential ones (Kimura and Ohta 1974). Advances in computational evolutionary modeling have yielded robust methods for estimating site-specific substitution rates from amino acid and DNA sequences (Yang 1994, 1996, Yang *et al.* 2000, Echave *et al.* 2016). Comparison of orthologous sequences (gene sequences in different species that have evolved from a common ancestor through speciation) reveals conserved amino acid sites that are likely to be functional and/or structurally important (Capra and Singh 2007). These amino acid sites tend to evolve under intense purifying selection to maintain protein function.

Several bioinformatics tools based on phylogenetics have been developed to identify functional amino acid sites (Pupko *et al.* 2002, Simon *et al.* 2002, Innis *et al.* 2004, Nimrod *et al.* 2005, Capra and Singh 2007). These tools have shown that conserved sites can align with experimental evidence, validating their utility despite a few limitations. For example, Rate4Site is widely used and provides valuable insights by estimating site-specific substitution rates based on a probabilistic framework (Pupko *et al.* 2002). Like many other methods, it assumes that substitution rates are independent across amino acid sites and identically distributed in the sequence alignment. While this assumption simplifies statistical modeling and enables efficient rate estimation, it does not fully capture the structural and functional dependencies among sites. In reality, functional amino acid sites tend to cluster within specific regions, such as binding or catalytically active sites (Madabushi *et al.* 2002, Panchenko *et al.* 2004). Consequently, incorporating structural context alongside phylogenetic conservation can further refine the identification of functional sites.

To address this limitation, some methods have been introduced to account for the spatial correlation of evolutionary patterns. Many of these methods use a sliding window framework that approximates the site-specific substitution rate for a given amino acid site by the average of neighboring sites within the protein tertiary structure (Suzuki 2004, Berglund *et al.* 2005, Watabe and Kishino 2013). An amino acid site is considered a neighbor if its Euclidean distance from the focal site is within a predefined window size. However, sliding window methods have inherent disadvantages. First, most methods give equal weight to the focal site and its neighbors, even though the focal site contains more pertinent information about its substitution rate. Second, determining the optimal window size is challenging. A window that is too large will include too many distant sites, potentially biasing the estimate of the target amino acid site, while a window that is too small will fail to capture spatial correlation and may lead to overfitting. In addition, there is evidence that the optimal window size may vary between different protein families (Suzuki 2004).

A relatively novel phylogenetic Gaussian process model has been developed to identify conserved amino acid patches in protein tertiary structure (Huang and Golding 2014). This approach estimates spatially correlated site-specific substitution rates by computing an optimal characteristic length, enabling the identification of functional and/or structurally important regions. The model, implemented in the GP4Rate software, leverages the Markov chain Monte Carlo method to generate samples, which, while computationally intensive (ranging from hours to days depending on dataset size), provides a robust probabilistic framework for detecting evolutionary constraints. To address computational efficiency, the FuncPatch web server was later developed as a faster approximation of GP4Rate (Huang and Golding 2015), making the method more accessible. In addition, GP4Rate evaluates spatial correlation based on Cα atoms, which effectively captures backbone geometry but does not explicitly consider side chain orientation. While this simplification is sufficient for many protein classes, incorporating side chain orientation could be particularly beneficial for helix-enriched proteins such as transmembrane proteins. Despite their potential, GP4Rate and FuncPatch are no longer available, highlighting the need for continued development of tools integrating both phylogenetic conservation and structural context. Very recently, another tool was developed, named xProtCAS, which integrates information on residue solvent accessibility and conservation with topological data on residue proximity in 3D space using graph-based methods to determine proximal clusters of relatively conserved residues on a protein’s surface (Kotb and Davey 2023). Although very useful, the tool is currently limited to the human proteome.

In this study, we developed an improved strategy called CONSTRUCT to identify patches of conserved sites within protein tertiary structures as putative functional and/or structurally important regions. Site-specific substitution rates are first calculated using the Rate4Site model and then weighted by the site-specific substitution rates of neighboring amino acid sites according to Euclidean distances, over a range of window sizes [from 1 to 20 Ångströms (Å)]. The optimal window size is determined by identifying the strongest spatial correlation of site-specific substitution rates, if present. The analysis can be performed either on Cα atoms or using the center of mass of amino acid sites as a proxy for side chain orientation, allowing for a more refined assessment of spatial constraints. To evaluate the accuracy of CONSTRUCT in identifying critical protein regions, we conducted extensive benchmarking through hundreds of simulations, and applied the method to 14 functionally characterized proteins, including transport and membrane proteins, enzymes, and oncoproteins. These case studies spanned a wide range of protein sizes, evolutionary conservation levels, and taxonomic groups (*Metazoa*, *Fungi*, *Chromalveolata*, *Bacteria*).

## 2 Materials and methods

### 2.1 Calculation of site-specific substitution rates

**Figure 1.**
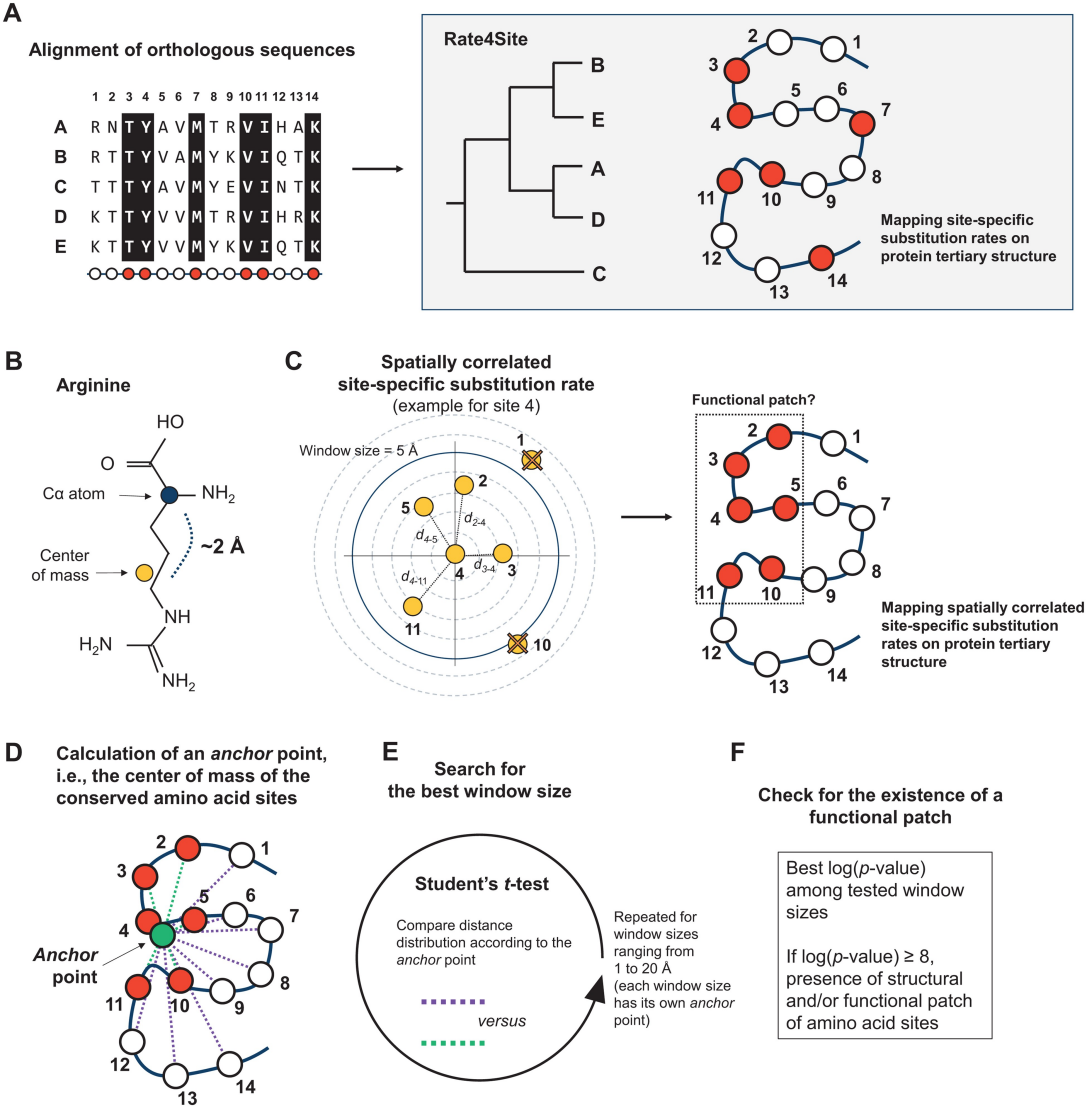
Overview of the CONSTRUCT algorithm. (A) CONSTRUCT requires a multiple orthologous sequence alignment and a representative tertiary structure. A phylogenetic tree is constructed to infer site-specific substitution rates using Rate4Site, which ignores the spatial correlation of site-specific substitution rates in protein tertiary structure. (B) The Euclidean distance between amino acid sites is calculated using either the Cα atoms or the center of mass of amino acids as a *proxy* for side chain orientation (here using an arginine as an example). (C) For an amino acid site, its site-specific substitution rate is weighted by the rates of its neighboring residues; this weighting is distance-dependent: the influence of a neighbor's substitution rate decreases as the Euclidean distance between residues increases. In this example, the spatially correlated site-specific substitution rate is calculated for site 4 in a window size of 5 Å (a range of distances from 1 to 20 Å is performed with a step size of 1 Å). The spatially correlated site-specific substitution rate is calculated for all sites, then the most conserved sites are highlighted on the protein tertiary structure. (D) One *anchor* point per protein is calculated as the geometric center of the most conserved sites of the protein. (E) A Student’s *t*-test is performed to compare the Euclidean distances of the most conserved amino acid sites to the *anchor* point with the Euclidean distances of the remaining amino acid sites to the *anchor* point. The proportion of the most conserved amino acid sites can be defined by the user (default: 10%). The analysis is performed for each window size (one anchor point is calculated for each window size). (F) A log(*P*-value) ≥8 indicates the presence of a spatial correlation in site-specific substitution rates. Only the results with the window size that maximizes the spatial correlation are kept.

The calculation of site-specific evolutionary rates is performed using the Rate4Site computational tool (Pupko *et al.* 2002) with an amino acid sequence alignment as input (Fig. 1A). The user must ensure the quality of the alignment and remove gapped and misaligned regions. Rate4Site infers a phylogenetic tree to model the evolutionary relationships among the sequences, which is used to weight the calculation of site-specific substitution rates. It assumes that the provided multiple sequence alignment represents orthologous sequences and that each site in the alignment corresponds to the same position in all sequences. The algorithm is implemented in a Bayesian framework and uses a random-effects approach, specifying gamma distribution as the prior rate distribution. An Empirical Bayes approach is then used to calculate site-specific substitution rates. The output is a score for each amino acid site, where lower scores indicate more conserved amino acid sites (likely under purifying selection) and higher scores indicate more variable amino acid sites. Note that the error rate associated to each amino acid site is ignored.

### 2.2 Estimation of spatially correlated site-specific substitution rates

Once the site-specific substitution rates have been estimated, these rates are further refined by incorporating the structural context of the protein. This process requires the user to provide a tertiary structure of the protein in PDB format. Note that the user can submit a protein tertiary structure retrieved from the AlphaFold database (Abramson *et al.* 2024). The reference sequence from the multiple sequence alignment and the sequence extracted from the PDB file are aligned to map the site-specific substitution rates to the corresponding amino acid sites in the tertiary structure.

After mapping the substitution rates to the structure, a predefined window size is used to identify neighboring amino acid sites for each residue in the protein. Two approaches can be used to define these neighbors: one based on the peptide backbone, specifically Cα atoms, and the other based on the center of mass of each amino acid site (Fig. 1B). The center of mass approach takes into account the spatial orientation of the amino acid side chains, providing a likely more accurate representation of the local structural environment.

For each amino acid site, its site-specific substitution rate is then weighted by the rates of its neighboring residues (Fig. 1C). This weighting is distance-dependent, meaning that the influence of a neighbor's substitution rate decreases as the Euclidean distance (expressed in Å) between the residues increases (1).

(1) $$\bar{S}(A)=\frac{1}{N}\sum_{i=1}^{N}\{\begin{array}{c} S_{i},\;if\;d_{i}=0\\ \frac{S_{i}}{d_{i}},\;\mathrm{else} \end{array}$$

where *A* is the amino acid site for which we want to calculate the spatially correlated site-specific substitution rate, *N* is the total number of amino acid sites within the predefined window size, *S_i_* is the site-specific substitution rate at the amino acid site *i* calculated with Rate4Site, and *di* is the Euclidean distance between two amino acid sites.

### 2.3 Detection of conserved amino acid patches

First, conserved amino acid patch(es) can be identified by eye by looking at the spatially correlated site-specific substitution rates mapped on the protein surface. Second, to investigate formally the existence of a conserved amino acid patch, we implemented a statistical approach. An artificial anchor point is generated within the 3D structure of the protein. This anchor point is defined as the center of mass of the amino acid sites with the lowest spatially correlated site-specific substitution rates (i.e. the geometric center of the most conserved amino acid sites of the protein) (Fig. 1D). By default, the anchor point is calculated using the 10% most conserved sites, although this parameter can be modified by the user. Next, the Euclidean distance (in Å) between the anchor point and each amino acid site (either the Cα atoms or the centers of mass, depending on the user choice) is calculated. These distances provide a measure of how spatially close is each amino acid site to the most conserved region of the protein.

The Euclidean distances to the anchor point of the most conserved amino acid sites are then compared to those of the remaining amino acid sites by a Student's *t*-test (Fig. 1E). A significant difference between these two sets of distances suggests a spatial clustering of conserved residues around the anchor point.

After performing the statistical test, the logarithm of the *P*-value obtained from the test is calculated. This log-transformed *P*-value provides a more interpretable measure of statistical significance. A log(*P*-value) ≥8 serves as a conservative threshold to confirm the existence of a spatial correlation in site-specific substitution rates (Fig. 1F). It also confirms the presence of a conserved amino acid patch that is likely to be critical for the function and/or structural integrity of the protein.

### 2.4 Determining the best window size for spatially correlated site-specific substitution rates

The strength of spatial correlation of site-specific substitution rates can vary among proteins and also depends on the window size chosen. Therefore, the calculation of site-specific substitution rates weighted by the 3D environment is performed over a range of distances from 1 to 20 Å, with a step size of 1 Å (Fig. 1E). The upper limit of 20 Å is based on the assumption that an amino acid site that is excessively distant from the residue under study may have a negligible effect on its evolutionary rate.

For each window size within this range, spatially correlated site-specific substitution rates are calculated. This results in 20 different sets of spatially correlated site-specific substitution rates. Distance comparisons are made between the most conserved amino acid sites or the less conserved amino acid sites and the anchor point, independently for each window size. The 20 log-transformed *P*-values are then calculated and compared. Among the 20 window sizes, the highest log(*P*-value) is considered the optimal value, as it represents the system that maximizes the strength of the spatial correlation. This approach ensures that the selected window size best captures the local structural influences on the evolutionary rates.

### 2.5 Justifying the fixed parameters through simulations

To demonstrate the relevance of the tools and threshold values set in our pipeline, we conducted an extensive series of simulations. The generation of simulated multiple sequence alignments was performed using the AliSim tool (Ly-Trong *et al.* 2022) implemented in IQ-TREE 2 (Minh *et al.* 2020).

The first series of simulations aimed to compare computation times and site-specific substitution rates between Rate4Site and CodeML. CodeML estimates site-specific substitution rates under codon-based models of evolution, taking into account synonymous and nonsynonymous substitution rates (*d*_N_/*d*_S_) to infer selective constraints at each codon position (Yang 2007). Unlike Rate4Site, which operates on amino acid alignments, CodeML requires a nucleotide alignment and a corresponding phylogenetic tree. Nucleotide alignments of varying lengths (200–300 nucleotides) and consisting of 10–30 sequences were generated. A substitution model MG was applied, with site heterogeneity rates following a gamma distribution with shape parameter ranging from 0.2 to 0.6. In total, 72 nucleotide alignments were generated, with a corresponding phylogenetic tree estimated using IQ-TREE 2. The nucleotide alignments were then translated into protein sequences before being processed with Rate4Site using a Python script.

The second series of simulations was designed to validate the application of our algorithm on large datasets, considering protein size, the number of sequences in the alignment, and interspecies conservation levels. Additionally, these simulations aimed to justify the selection of the 10% threshold for identifying conserved amino acid patches and to establish the log(*P*-value) ≥ 8 threshold as highly conservative. In total, 1000 multiple sequence alignments were generated using AliSim under the LG substitution model, with site heterogeneity rates following a gamma distribution with shape parameter between 0.5 and 0.9. Each dataset was randomly composed of 20–200 amino acid sequences. Five groups of 200 alignments were generated, according to protein length: 50–100 amino acids, 100–200 amino acids, 200–300 amino acids, 300–400 amino acids, and 400–500 amino acids. Rate4Site was then applied to each of the 1000 alignments. The first sequence of each alignment was considered as the reference and used to produce a random 3D structure. The amino acid sites were spaced at an average of approximately 3.8 Å, ensuring that 30% of the most conserved amino acids were spatially clustered in the structure. This setup was designed to create a “control” structure where a conserved amino acid patch should be detectable, with the 30% threshold chosen to ensure the patch was not always easily identifiable. Once the alignments and structures were generated, our software was applied in different contexts:

1. In standard mode to verify the expected presence of a conserved amino acid patch.
2. In standard mode while varying the proportion of the most conserved sites (used to fix the anchor point), ranging from 5% to 25%, to demonstrate that the 10% threshold is the most suitable (while remaining user-configurable).
3. By performing random permutations (100 replicates per dataset) of the site-specific substitution rates to theoretically remove the conserved amino acid patch, demonstrating that the log(*P*-value) ≥ 8 threshold is highly conservative

### 2.6 Application of CONSTRUCT to case studies

All of the tasks described above have been integrated into a software package called CONSTRUCT. To demonstrate the accuracy of CONSTRUCT in identifying functional amino acid patches, we developed a series of datasets for several functionally characterized proteins, including transport and membrane proteins, enzymes, and oncoproteins. These proteins varied in size, conservation levels across species, and originate from different kingdoms/domains including mammals, parasites, and bacteria (Table 1). Here we focused on cytochrome c, E3 ubiquitin-protein ligase MDM2, GTPase HRas, myoglobin, cystic fibrosis transmembrane conductance regulator (CFTR), cAMP-dependent protein kinase catalytic subunit alpha, mitogen-activated protein kinase 1, sodium/glucose cotransporter 1 (SLGT1), Kelch-like ECH-associated protein 1 (KEAP1), and torsin-1B in mammals; the dihydropteroate synthase (DHPS) and dihydrofolate reductase (DHFR) enzymes in unicellular parasites; the aromatic amino acid exporter YddG in bacteria; and the GDP-mannose transporter 1 in fungi. For each protein, detailed information was compiled, including its function, cellular localization, conserved domains, amino acid sites reported to be functional, and a description of its 3D structure (Supplementary Data S1). For torsin-1B, the 3D structure was not resolved and was predicted using the AlphaFold 3 algorithm (DeepMind, Google) (Abramson *et al.* 2024) to show that CONSTRUCT can also identify relevant functional regions with predicted structures. All results were compared with those of (i) Rate4Site, which ignores the spatial correlation of site-specific substitution rates in protein tertiary structure (Pupko *et al.* 2002); and (ii) xProtCAS, which searches for patches of conserved amino acid sites at the protein surface (only for human proteins) (Kotb and Davey 2023). Unless otherwise specified, the calculation of spatially correlated site-specific substitution rates was based on the center of mass of amino acid sites as Cartesian coordinates. The execution time of the software was benchmarked across the case studies on a Linux system (Ubuntu 22.04.4 LTS) consisting of 16 Go random access memory (RAM) and an Intel Core I7-6820HQ (Supplementary Table S1).

**Table 1.** Characteristics of the proteins investigated in this study.

Protein	Classification	Main function	Kingdom or class	Reference species	Uniprot ID	Protein length	Protein domain investigated	PDB structure	No. of orthologous sequences	Alignment length	Conservation level among orthologous (%)
Cytochrome c	Transport protein	Electron transfer	*Metazoa*	*Homo sapiens*	P99999	105		1J3S	853	103	85.15
MDM2	Oncoprotein	Regulates p53	*Metazoa*	*H. sapiens*	Q00987	491	N-terminal domain (25–109)	1YCR	376	109	92.10
KEAP1	Peptide binding protein	Regulates Nrf2	*Metazoa*	*H. sapiens*	Q14145	624	Kelch domain (325–609)	2FLU	135	285	87.89
Myoglobin	Transport protein	Storage and transport of oxygen	*Metazoa*	*H. sapiens*	P02144	154		3RGK	454	153	86.83
CFTR	Membrane transport protein	Chloride ion transport, regulation of salt and water transport	*Metazoa*	*H. sapiens*	P13569	1480		8FZQ	415	1463	88.47
cAMP-dependent protein kinase A	Transferase	Phosphorylation of serine and threonine on target proteins	*Metazoa*	*H. sapiens*	P17612	351		4WB5	254	334	97.53
DHFR	Oxidoreductase	Catalyzes the reduction of dihydrofolate to tetrahydrofolate	*Chrom-alveolata*	*Plasmodium falciparum*	A7UD81	608	N-terminal domain (1–231)	3QGT	366	617	48.53
DHPS	Transferase	Catalyzes the formation of dihydropteroate	*Chrom-alveolata*	*P. falciparum*	Q25704	706	Catalytic domain (383–708)	6JWV	55	641	64.15
Mitogen-activated protein kinase 1	Transferase	Transmits extracellular signals to the nucleus	*Metazoa*	*Rattus norvegicus*	P63086	358		5UMO	493	365	73.93
SGLT1	Membrane transport protein	Glucose and galactose transport	*Metazoa*	*H. sapiens*	P13866	664		7SL8	402	667	79.34
Torsin-1B	Chaperone protein	Participates in protein folding	*Metazoa*	*H. sapiens*	O14657	336		Predicted	299	338	81.08
YddG	Membrane transport protein	Transport of aromatic amino acids	*Bacteria*	*Ancylobacter novellus*	D7A5Q8	287		5I20	383	277	63.47
GDP-mannose transporter 1	Membrane transport protein	GDP-mannose transport	*Fungi*	*Saccharomyces cerevisiae*	P40107	337		5OGE	366	322	66.53
HRas	Oncoprotein	Ras protein signal transduction	*Metazoa*	*H. sapiens*	P01112	189		5P21	421	166	93.62

### 2.7 Implementation

CONSTRUCT can be run on any computer with a Linux or macOS system and the Python 3 and R programming languages installed. The software can be easily installed using a bash script that checks for dependencies (Supplementary Table S2). To simplify the use of CONSTRUCT, a graphical user interface has been developed. The CONSTRUCT program and documentation are freely available on GitHub (https://github.com/Rcoppee/CONSTRUCT). A video tutorial has been created for the installation and use of CONSTRUCT: https://youtu.be/bf-VYReZIeM.

## 3 Results

### 3.1 Computation choices and parameters implemented in CONSTRUCT

To justify the parameters and computational choices implemented in CONSTRUCT, we conducted an extensive series of simulations. These analyses aimed to validate key methodological decisions, including the selection of site-specific substitution rate estimation methods, statistical thresholds, and the proportion of conserved amino acid sites used for patch detection.

**Figure 2.**
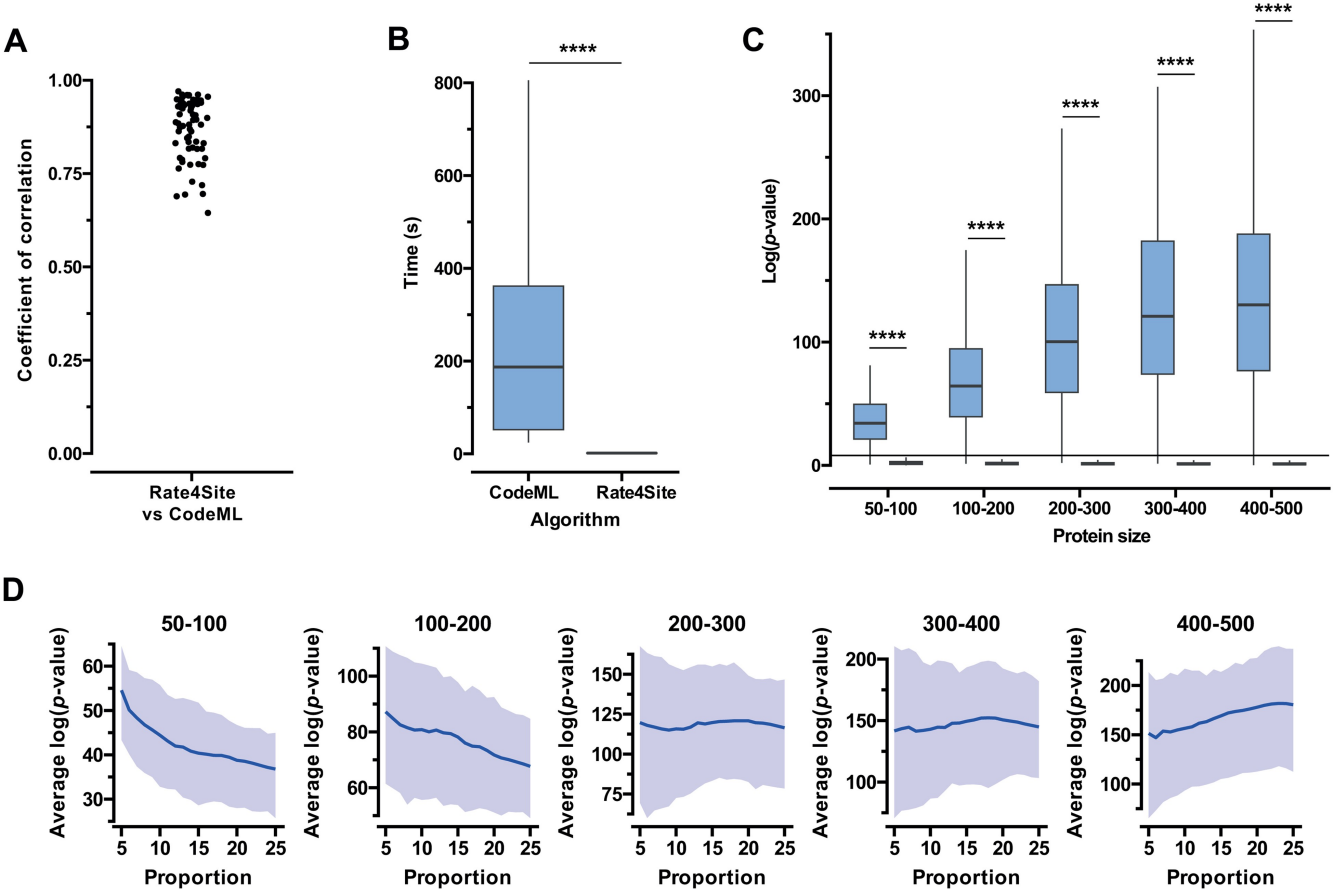
Simulation results evaluating the tools and parameters used in CONSTRUCT. (A) Spearman’s correlation results of site-specific substitution rates estimated by Rate4Site and CodeML across 72 simulated alignments (*r* = 0.87 on average). (B) Computational time comparison for site-specific substitution rate estimation using Rate4Site or CodeML. (C) Distribution of log(*P*-values) for the detection of patch of conserved amino acid sites across different protein sizes. The results included 200 simulations for each group of protein size. Boxplots on the left correspond to positive controls, where a patch of conserved amino acid sites is expected to be detected, while those on the right represent negative controls, where site-specific substitution rates were aleatory permuted to remove the putative presence of conserved patch. The black line indicates the significance threshold at log(*P*-value) = 8, above which patches are considered statistically significant. ****, *P*-value < 0.0001 (Mann–Whitney *U* test). (D) Impact of the proportion of conserved amino acid sites on patch detection across different protein sizes. Results are based on the same simulations used in (C). The blue curve represents the mean log(*P*-value), while the shaded blue region indicates the interquartile range (first to third quartile) across simulations.

As a first step, we assessed the reliability and efficiency of site-specific substitution rates obtained from two widely used phylogenetic approaches: Rate4Site (at the protein level) and CodeML (at the codon level). The results showed very similar site-specific substitution rates inferred by both methods (Spearman’s correlation coefficient of *r* = 0.87 on average) in the tested simulations (Fig. 2A). However, in terms of computational efficiency, Rate4Site is significantly faster, with an average execution time of 1.6 s per simulation, compared to 218.9 s for CodeML under identical conditions (Fig. 2B). This ∼137-fold speed improvement makes Rate4Site a more practical choice for large-scale or high-throughput analyses, without compromising the reliability of site-specific substitution rate estimation. Given these results, we chose to implement Rate4Site in CONSTRUCT as the default method for estimating site-specific substitution rates.

To rigorously justify the threshold of log(*P*-value) ≥ 8 used in CONSTRUCT for detecting conserved amino acid patches, we generated 1000 multiple sequence alignments featuring varying protein sizes (ranging from 50 to 500 amino acids), different numbers of sequences (between 20 and 200), and heterogeneous conservation levels. For each alignment, a corresponding 3D protein structure was randomly generated, simulating realistic spatial arrangements, and ensuring that 30% of the most conserved amino acids were spatially clustered. Across all tested proteins, regardless of their size, we consistently detected a patch of conserved amino acid sites in the vast majority of cases, as expected [98.0% of cases with log(*P*-value ≥ 8); Fig. 2C and Supplementary Table S3]. We observed a positive correlation between the log(*P*-value) and protein size, aligning with theoretical expectations: larger proteins provide a greater number of amino acids, enhancing statistical power and improving the ability to discriminate functionally constrained regions. To further challenge this threshold, we performed a series of random permutations—100 independent permutations for each of the 1000 dataset—effectively removing any biologically meaningful clustering of conserved residues. In these randomized scenarios, the log(*P*-value) remained below 8 in nearly all cases (98.7%), irrespective of protein size (Supplementary Table S4). These findings strongly support that a log(*P*-value) ≥ 8 is a highly conservative threshold for the detection of a significant conserved patch. To validate this further, we applied this threshold to two independent case studies, both of which yielded identical conclusions (Supplementary Fig. S1). Altogether, both simulations and case studies confirmed that the threshold is effective and stringent, providing reliable detection of conserved regions across diverse protein sizes and conservation landscapes.

We finally extended the 1000 simulations described previously, this time by systematically varying the proportion of the most conserved amino acid sites used for patch identification. Specifically, we tested thresholds ranging from 5% to 25%, while keeping all other parameters identical (protein size, number of sequences, and conservation heterogeneity). As previously, we identified a protein size effect when modifying the proportion of conserved residues used for patch detection (Fig. 2D). For small proteins (50–100 residues), selecting only 5% of the most conserved residues resulted in a higher log(*P*-value), but at the cost of including as few as 2–5 amino acid sites, which could lead to patches too small to be biologically meaningful. Conversely, for larger proteins (>400 residues), selecting 25% of the most conserved residues resulted in a higher log(*P*-value) as well, but at the cost of incorporating too many residues, leading to patches that are too large and likely include false positive functional sites. Despite these variations, we observed that the log(*P*-value) remained consistently above 8 across all conditions, confirming that patch detection remains statistically robust regardless of the proportion of selected residues. However, balancing sensitivity and specificity is essential: while increasing the proportion of selected residues improves statistical power in large proteins, an excessive number of amino acid sites dilutes the signal and increases the risk of including nonfunctional sites and vice versa. Given these findings, the 10% threshold emerges as the most balanced and operational choice, ensuring that patches are representative without being overly inclusive. This threshold aligns with previous studies in protein evolution and structural biology, where a similar 10% cutoff has been used to define functionally constrained sites (Huang and Golding 2014, 2015).

### 3.2 CONSTRUCT identifies functional amino acid patches

We then applied CONSTRUCT to 14 functionally characterized proteins. Here, we focus on two case studies: the CFTR protein, which is responsible for chloride ion flux and whose mutations cause cystic fibrosis (Devidas and Guggino 1997), and the transmembrane protein YddG, which is found in bacteria and is involved in the export of aromatic amino acids (Tsuchiya *et al.* 2016). The results for the other case studies are described in the Supplementary Material (Supplementary Data S2).

**Figure 3.**
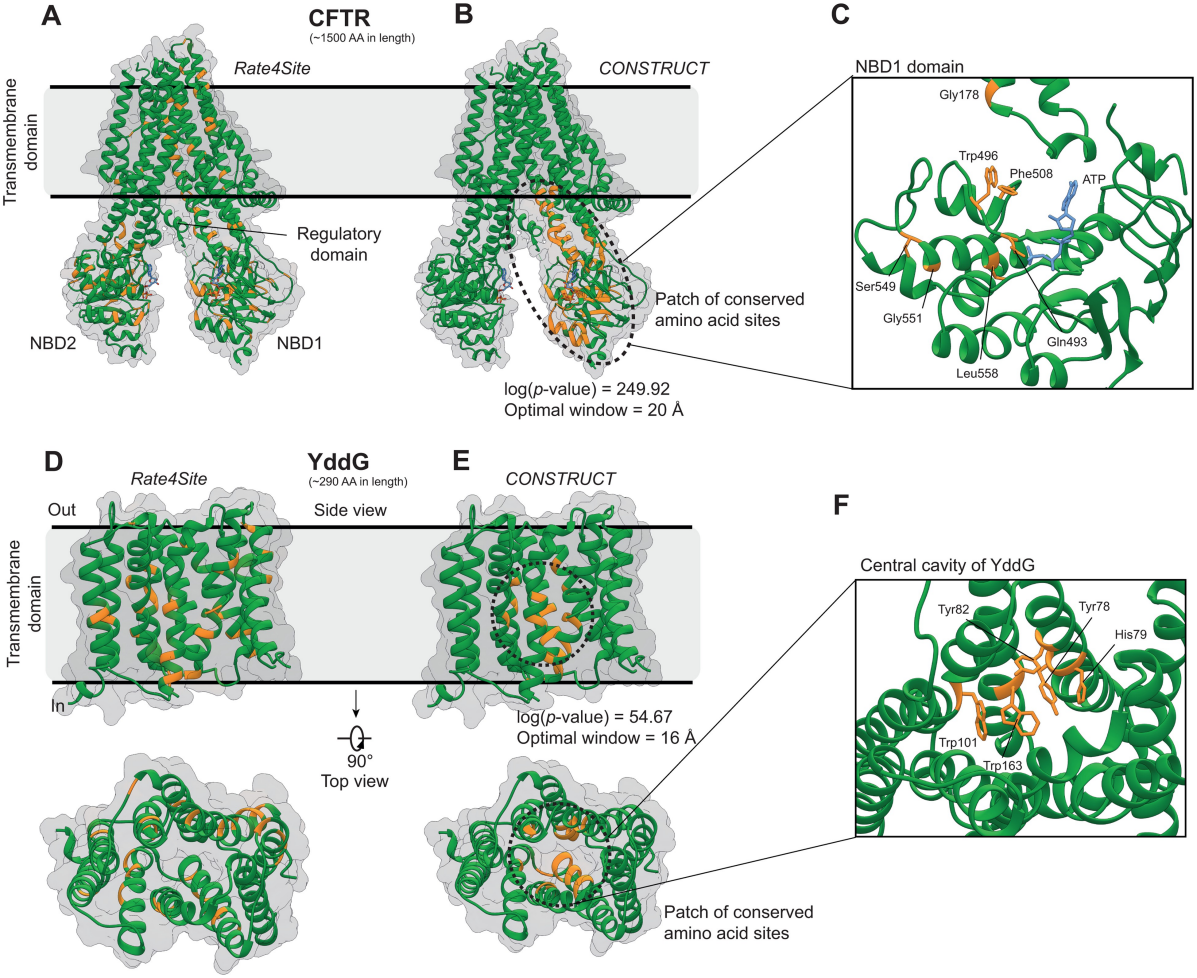
Identification of patches of functionally validated amino acid sites in CFTR and YddG proteins. Location of the 10% most conserved amino acid sites (colored in orange) in the tertiary structure of CFTR and YddG according to (A and D) Rate4Site and (B and E) CONSTRUCT. The functional domains of CFTR and YddG are annotated. (C and F) Zoom on some amino acid sites in the NBD1 domain of CFTR (which are essential for ATP hydrolysis or cause cystic fibrosis) and in the central channel of YddG (which are reported to be critical for transport activities), which belong to the patches of conserved amino acid sites detected by CONSTRUCT.

Rate4Site was first run on a dataset of 351 CFTR orthologous sequences and revealed that the most conserved amino acid sites were uniformly distributed throughout the tertiary structure of CFTR (Fig. 3A). Of note, these conserved sites included Gly178, Ser549, and Gly551, which are targets of ivacaftor (used to treat cystic fibrosis) (Yarlagadda *et al.* 2012, Zhang *et al.* 2018, Levring *et al.* 2023), Gln493, which is thought to be important for ATP hydrolysis (Lewis *et al.* 2005), and Phe508, which is associated with the major mutation that confers cystic fibrosis (Phe508 deletion) (Lewis *et al.* 2005). CONSTRUCT was then run on the same dataset. A patch of conserved amino acid sites was detected [log(*P*-value) = 249.92] at an optimal distance of 20 Å (Table 2). This patch covered a large part of one of the two nucleotide-binding domains (NBD), namely NBD1, critical for regulation of channel activity and binding and hydrolysis of ATP (Fig. 3B and C) (Duffieux *et al.* 2000). The patch included the functional amino acid sites mentioned above, but also Trp496, which is close to Phe508 and reduces the amount of CFTR when mutated to arginine (Thibodeau *et al.* 2005), and Leu558, which induces delayed compaction of nascent NBD1 when mutated to serine (Shishido *et al.* 2020). We next focused on YddG to test whether CONSTRUCT is able to capture patches of conserved amino acid sites in the context of transporter proteins. Here we used a dataset consisting of 366 YddG orthologous sequences. Again, the most conserved amino acid sites detected by Rate4Site were distributed throughout the protein structure (Fig. 3D). A few amino acid sites were located in the channel of the transporter, and only Trp101—reported to be important for transport activity—had its side chain oriented toward the channel (Tsuchiya *et al.* 2016). CONSTRUCT identified a patch of conserved amino acid sites in the core of the transporter [log(*P*-value) = 54.67] at an optimal distance of 16 Å (Fig. 3E and Table 2). The patch included Trp101, but also Tyr78, His79, Tyr82, and Trp163 (Fig. 3F), all of which have been reported to be critical for transport activities as evidenced by site-directed mutagenesis (Tsuchiya *et al.* 2016). In other case studies, a patch of conserved amino acid sites was systematically detected, in contrast to the broad distribution of conserved amino acid sites proposed by Rate4Site alone (Table 2 and Supplementary Data S2). This included the torsin-1B case where the protein tertiary structure was not experimentally determined but instead predicted using AlphaFold. All detected patches for each protein were strongly associated with experimentally verified functional regions. For human proteins (*n* = 9), the patches of conserved amino acid sites aligned with those detected using the xProtCAS tool in seven cases (Supplementary Fig. S2). Overall, the results showed that CONSTRUCT can effectively identify patches of amino acid sites associated with functional constraints.

**Table 2.** Results of CONSTRUCT for each case study.

Protein	Coordinates	Patch detected	log(*P*-value)	Optimal length (A)	Signification of the patch
Cytochrome c	Cα	Yes	23.97	12	Binding site with heme molecule
	Side chain	Yes	15.99	13	
MDM2	Cα	Yes	15.82	9	Partially overlaps with p53 interaction surface
	Side chain	Yes	15.07	10	
KEAP1 (propeller)	Cα	Yes	65.43	19	Interaction surface with Nrf2
	Side chain	Yes	74.63	14	
Myoglobin	Cα	Yes	48.01	17	Interaction surface with Cu(II)
	Side chain	Yes	41.86	20	
CFTR	Cα	Yes	285.39	19	NBD1 domain, including several amino acid sites targeted by drugs
	Side chain	Yes	249.92	20	
cAMP-dependent protein kinase A	Cα	Yes	62.17	14	Active kinase core of the protein
	Side chain	Yes	78.87	17	
DHFR	Cα	Yes	71.69	15	Binding site with NADPH (and pyrimethamine antimalarial drug)
	Side chain	Yes	72.82	16	
DHPS	Cα	Yes	66.32	10	Binding site with sulfa derivatives (such as sulfadoxine)
	Side chain	Yes	60.62	19	
Mitogen-activated protein kinase 1	Cα	Yes	60.67	16	Catalytic site of the protein
	Side chain	Yes	50.73	17	
SGLT1	Cα	Yes	74.46	20	Binding site with glucose
	Side chain	Yes	88.76	20	
Torsin-1B	Cα	Yes	122.16	20	Binding site with ATP
	Side chain	Yes	106.53	19	
YddG	Cα	Yes	69.60	14	Core of the transporter, binding site with aromatic amino acids
	Side chain	Yes	54.67	16	
GDP-mannose transporter 1	Cα	Yes	66.20	17	Core of the transporter, binding site with GMP
	Side chain	Yes	54.76	8	
HRas	Cα	Yes	36.36	16	Biding site with its effectors
	Side chain	Yes	29.44	17	

### 3.3 Comparison between Cα atoms and the center of mass of amino acids as input coordinates

**Figure 4.**
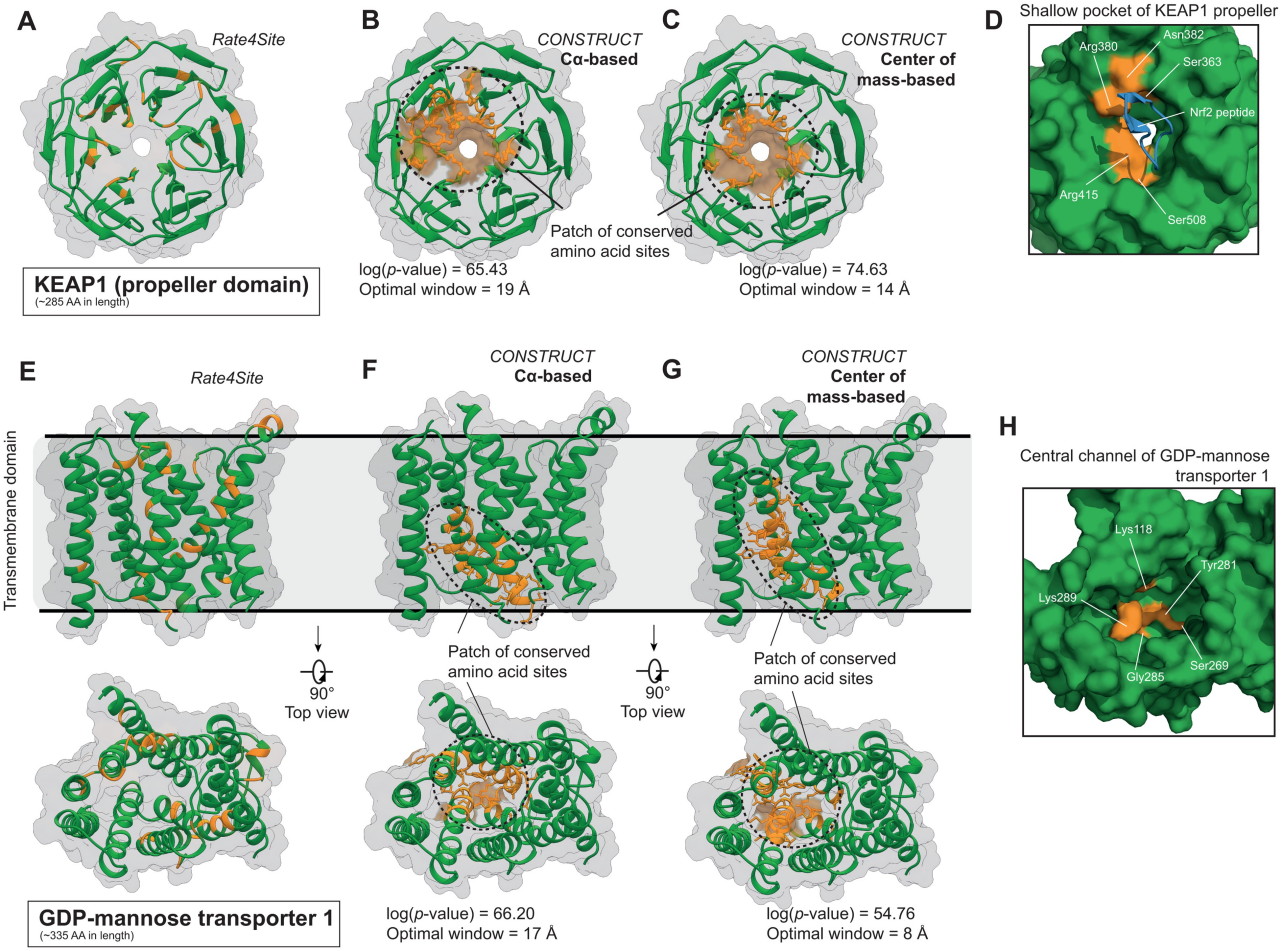
Comparison of functional patch delineation using either Cα atoms or center of mass of amino acid sites as input coordinates. Location of the 10% most conserved amino acid sites (colored in orange) in the tertiary structure of KEAP1 propeller and GDP-mannose transporter 1 according to (A and E) Rate4Site and CONSTRUCT using either (B and F) the Cα atoms or (C and G) the center of mass of amino acid sites as input coordinates. (D and H) Zoom on some amino acid sites in the shallow pocket of KEAP1 propeller [which interacts with Nrf2 (shown as cartoon and colored in blue)] and in the central channel of GDP-mannose transporter 1 (which is reported to be critical for transport activities) that belong to the patches of conserved amino acid sites detected by CONSTRUCT.

Finally, we aimed to compare the delineation of conserved amino acid patches using either the Cα atoms or the center of mass of the amino acids (taking into account the side chain orientation) for a given protein. Here we illustrate the differences obtained for the Kelch-repeat propeller domain of the KEAP1 protein, which is involved in ubiquitination complexes and regulates the cellular level of its substrate Nrf2 (Canning *et al.* 2015), and the GDP-mannose transporter 1 membrane protein, which is essential for the transport of GDP-mannose from the cytosol to the lumen of the Golgi apparatus (Abe *et al.* 1999). Application of the Rate4Site algorithm revealed that conserved amino acid sites are uniformly distributed across the 3D structures of the KEAP1 propeller and GDP-mannose transporter 1 (Fig. 4A and E), based on 135 and 366 orthologous sequences, respectively.

CONSTRUCT was then applied in parallel using either the Cα atoms or the center of mass of the amino acids. For KEAP1, both strategies identified a conserved patch at the shallow pocket on the surface of the propeller domain, which was experimentally shown to be the interaction surface with Nrf2. Based on the Cα atoms, the patch was delineated with an optimal distance of 19 Å and a log(*P*-value) of 65.43 (Fig. 4B and Table 2). Four amino acids reported to be essential for interaction with Nrf2 were captured: Ser363, Arg380, Asn382, and Arg415 (Canning *et al.* 2015). Using the center of mass of the amino acids, the patch was delineated with an optimal distance of 14 Å and a log(*P*-value) of 74.63 (Fig. 4C and Table 2). Thus, the correlation strength was stronger when based on centers of mass. In addition, the mapping of the most conserved amino acid sites onto the structure revealed a better capture of the shallow pocket compared to the data obtained with the Cα atoms. Furthermore, in addition to the four amino acids mentioned above, we also captured Ser508, which is also reported to be important for the interaction with Nrf2 (Canning *et al.* 2015) (Fig. 4D).

The same strategy was applied to the GDP-mannose transporter 1 protein. Using both the Cα atoms and the centers of mass, a conserved amino acid patch was observed within the transporter channel, indicating the interaction zone with the transporter's substrate molecule. With the Cα atoms, the patch was delineated with an optimal distance of 17 Å and a log(*P*-value) of 66.20 (Fig. 4F and Table 2). Two amino acid sites important for the interaction with GDP-mannose or GMP substrates were located within this patch: Tyr281 and Gly285 (Parker and Newstead 2017). In terms of centers of mass, the patch was delineated with an optimal distance of 8 Å and a log(*P*-value) of 54.76 (Fig. 4G and Table 2). Although the correlation strength was a bit lower, the optimal distance was significantly shorter than for the Cα atoms. This patch included the two previously mentioned amino acid sites as well as Lys118, Lys289, and Ser269, which are also involved in the interaction with GDP-mannose or GMP (Parker and Newstead 2017) (Fig. 4H).

Taken together, these results showed that including the orientation of amino acid side chains in the search for conserved amino acid patches is complementary to the Cα-based analysis and can even improve the delineation of patches in specific structural contexts.

## 4 Discussion

Positive selection and purifying selection are two evolutionary mechanisms that affect the frequency of mutations in populations (Zhang and Yang 2015). Positive selection favors beneficial mutations that provide an adaptive advantage to the organism, thereby increasing their frequency over time (Zhang and Yang 2015, Echave *et al.* 2016). This process often leads to the emergence of new functions or the improvement of existing ones. In contrast, purifying selection eliminates deleterious mutations that could harm the organism’s survival or reproduction (Zhang and Yang 2015, Echave *et al.* 2016). This mechanism helps to maintain essential functions. Consequently, purifying selection is particularly important for identifying functional regions within proteins, because sites under strong purifying selection are usually critical for the function or structure of the protein (Pupko *et al.* 2002, Echave *et al.* 2016).

Many phylogenetic methods have been developed to identify slowly evolving amino acid sites (Pupko *et al.* 2002, Simon *et al.* 2002, Innis *et al.* 2004, Nimrod *et al.* 2005, Capra and Singh 2007). However, most approaches ignore the spatial correlation of site-specific substitution rates, or use only the polypeptide backbone to search for spatial correlation. We therefore developed CONSTRUCT, an intuitive software with a graphical interface to make it accessible to biologists and researchers not familiar with bioinformatics. Users only need to provide an alignment of orthologous sequences and a corresponding 3D structure in PDB format. The software is based on a simple strategy, which explains the relatively short computation times. Site-specific substitution rates are first calculated using the Rate4Site strategy and then weighted according to their spatial environment. Unlike most other approaches, the distance that maximizes the spatial correlation of site-specific substitution rates is determined directly by calculating all possible spatially correlated site-specific substitution rates for an amino acid site at a distance between 1 and 20 Å. By defining the center of mass of the most conserved amino acid sites as the focal point, a simple comparison of the Euclidean distances from this point to the most conserved amino acid sites and to the “less conserved” ones can confirm the existence of a cluster of conserved amino acid sites. Through a random permutation approach of the site-specific substitution rates, designed to remove the spatial correlation of the rates, we have defined a very conservative threshold beyond which a patch of conserved amino acid sites is considered to be present, regardless of protein size.

Through an extensive series of simulations and 14 case studies covering a wide range of proteins varying in size, structural complexity, and interspecies conservation levels, we showed CONSTRUCT’s ability to identify patches of amino acid sites critical for protein function and/or structure. Across all tested proteins, we consistently observed that experimentally validated functional amino acid sites were captured within the detected patches, reinforcing the biological relevance of our approach. These results confirm that CONSTRUCT can serve as a powerful tool for guiding site-directed mutagenesis experiments aimed at elucidating protein function. With the increasing role of artificial intelligence in structural biology, we further confirmed that CONSTRUCT performs robustly even when using structures predicted by AlphaFold (DeepMind, Google) (Abramson *et al.* 2024). Finally, we showed that incorporating side chain orientation—by defining the center of mass of amino acid sites rather than relying solely on the peptide backbone—can influence the delineation of conserved patches for certain 3D folds, as observed for the propeller domain of KEAP1 and within the GDP-mannose transporter 1 membrane transporter. While the strategies can be complementary, we believe that considering side chain orientation in the calculation of spatially correlated site-specific substitution rates better reflects the evolutionary dynamics of proteins, as previously proposed (Marcos and Echave 2015).

However, CONSTRUCT presents some limitations. First, if a protein contains multiple structural domains, it should be analyzed at the domain level rather than as a whole. When running CONSTRUCT on such a protein, each structural domain is expected to have its own conserved amino acid patch. Since the program calculates a single focal point (the center of mass of the most conserved amino acid sites), applying it to an entire multi-domain protein could lead to statistical biases by artificially grouping functionally unrelated residues. To address this, we have implemented a boundary system that allows users to specify the coordinates of individual domains, ensuring independent analyses. Similarly, the tool is not designed to analyze oligomers composed of multiple structural domains. Another important consideration is the proportion of highly conserved amino acid sites selected for patch detection. Our approach uses a default threshold of 10% (providing a good balance between sensitivity and specificity), which was determined through extensive simulations assessing statistical robustness across proteins of varying sizes and conservation patterns. Lower thresholds (e.g. 5%) may capture too few residues in small proteins, reducing statistical power, while higher thresholds (e.g. 25%) in large proteins can lead to overly diffuse patches, increasing false positives. Although this parameter can be changed by the user, a potential future improvement would be to implement an adaptive method based on protein structural families. Finally, our approach does not search for isolated functional sites, including post-translational modification sites or specific regulatory elements. Therefore, CONSTRUCT must be considered as a complementary tool to other methods such as Rate4Site.

In conclusion, CONSTRUCT is a simple and intuitive software that allows the determination of regions within a protein that are subject to strong purifying selection, often related to the physiological function of the protein. It can also suggest candidate amino acid sites for functional elucidation via site-directed mutagenesis approaches.

## Supplementary Material

btaf166_Supplementary_Data

## Data Availability

CONSTRUCT and documentation are freely available at https://github.com/Rcoppee/CONSTRUCT.
