# Supplementary material for: CONSTRUCT: an algorithmic tool for identifying functional or structurally important regions in protein tertiary structure
Source: Bioinformatics. 2025 Apr 12;41(4):btaf166. doi: 10.1093/bioinformatics/btaf166 (PMC12034385; doi:10.1093/bioinformatics/btaf166)
Supplement: btaf166_Supplementary_Data [file btaf166_supplementary_data.zip › supplementary_figures.pdf]

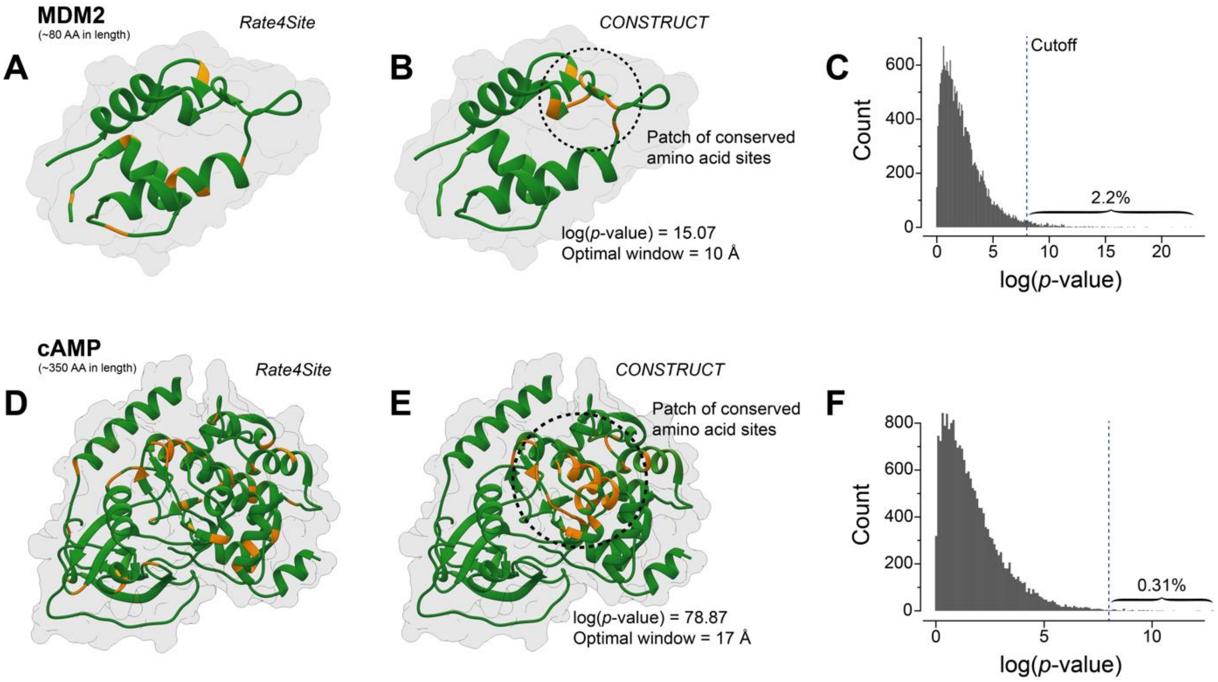

Supplementary Figure S1 – Defining the cutoff to detect patches of conserved amino acid sites. Location of the 10% most conserved amino acid sites (colored in orange) in the tertiary structure of MDM2 and cAMP according to **(A** and **D)** Rate4Site and **(B** and **E)** CONSTRUCT. **(C** and **F)** Distribution of the log(*p*-value) based on 1,000 independent replicates of the permutation analysis for MDM2 and cAMP, respectively. The cutoff of 8 is indicated by the dashed blue line. Permutations of the site-specific substitution rates resulted in an exponential decay of the log(*p*-value). We observed that only 2.2% and 0.31% of the permuted datasets were associated with a log(*p*-value) ≥ 8 for MDM2 and cAMP, respectively, confirming that the threshold of 8 is very conservative. This stringent threshold ensures the reliability of detecting true spatial correlations in site-specific substitution rates that indicate functional or structurally important amino acid patches.

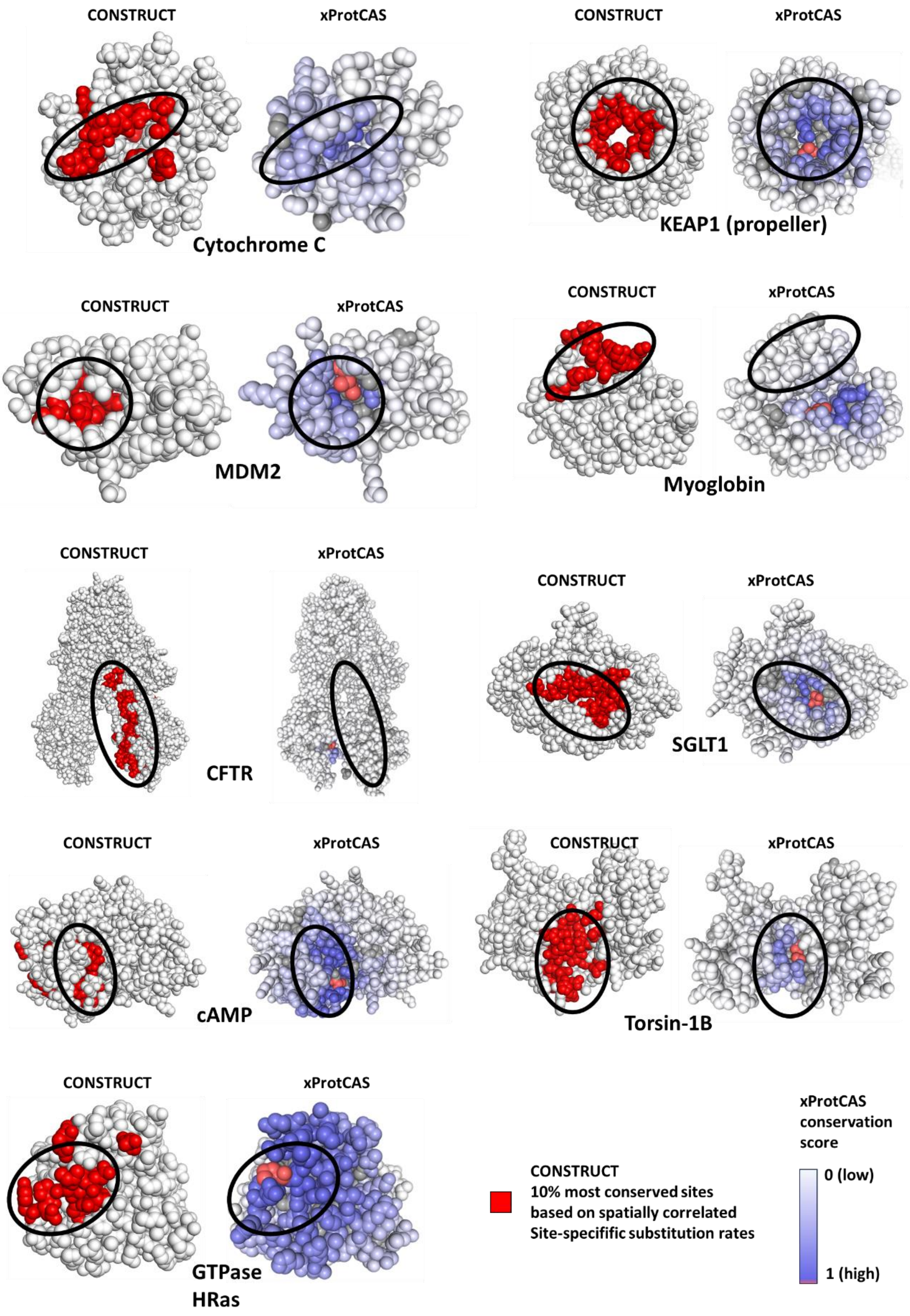

Supplementary Figure S2 – Comparison of conserved amino acid patches identified by CONSTRUCT and xProtCAS. Protein structures are displayed in ball representation to highlight individual amino acid sites. The conserved amino acid patch identified by CONSTRUCT is shown in red, while the conserved regions detected by xProtCAS are represented using a blue-to-red gradient, with red representing the most conserved site.
