# Supplementary material for: CONSTRUCT: an algorithmic tool for identifying functional or structurally important regions in protein tertiary structure
Source: Bioinformatics. 2025 Apr 12;41(4):btaf166. doi: 10.1093/bioinformatics/btaf166 (PMC12034385; doi:10.1093/bioinformatics/btaf166)
Supplement: btaf166_Supplementary_Data [file btaf166_supplementary_data.zip › supplementary_data_2.pdf]

Supplementary Data S2 – Detailed results of CONSTRUCT for other case studies

Cytochrome c (Uniprot: P99999, PDB ID: 1J3S)

Rate4Site was first run on a dataset of 853 Cytochrome c orthologous sequences. The most conserved amino acid sites were uniformly distributed throughout the tertiary structure of Cytochrome c (*left structure*). Of note, these conserved sites included Lys13, which is located in the heme binding site (1). CONSTRUCT was then run on the same dataset. A patch of conserved amino acid sites was detected (log(*p*-value) = 15.99) at an optimal distance of 13 Å. This patch covered the heme binding site (*right structure*), and contained Lys13, but also Gln16, His18, Lys79 and Ile81, which coordinate the heme iron and are important for the electron transfer function (1–4).

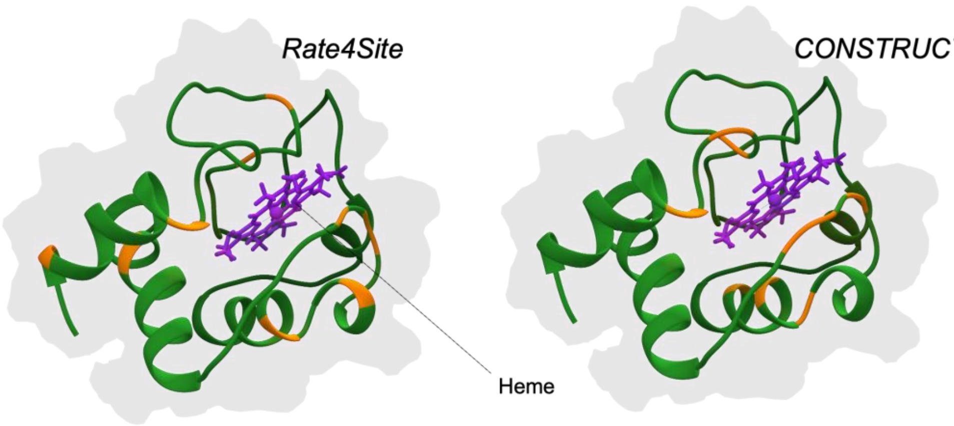

DHFR (Uniprot: A7UD81, PDB ID: 3QGT)

Rate4Site was first run on a dataset of 366 DHFR orthologous sequences. Again, the most conserved amino acid sites were uniformly distributed throughout the tertiary structure (*left structure*). These conserved sites included Ala16, which is associated with pyrimethamine resistance when mutated to valine and is located in the binding pocket with NADPH or pyrimethamine (5, 6). CONSTRUCT was then run on the same dataset. A patch of conserved amino acid sites was detected (log(*p*-value) = 72.82) at an optimal distance of 16 Å. This patch covered the binding site with NADPH or antimalarial drugs such as pyrimethamine (*right structure*). Especially, the patch included Ser108 and Ile112, both of which are located in the binding pocket and are involved in NADPH/pyrimethamine interaction (6, 7).

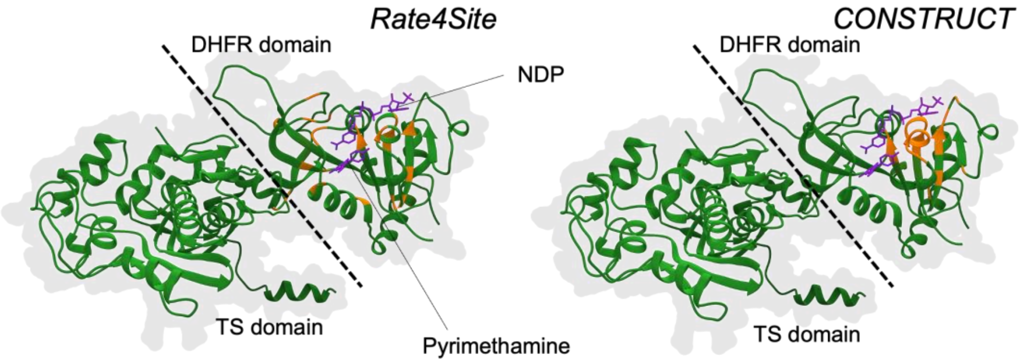

Myoglobin (Uniprot: P02144, PDB ID: 3RGK)
Using the Rate4Site algorithm, which ignores the spatial correlation of site-specific substitution rates in protein tertiary structure, conserved amino acid sites were widely distributed throughout the protein structure based on 454 orthologous sequences (*left structure*). Using CONSTRUCT, a spatial correlation of site-specific substitution rates was detected: the maximum strength of spatial correlation was observed at a distance of 20 Å, associated with a log(*p*-value) of 41.86. The most conserved sites identified by CONSTRUCT formed a well-defined patch in the tertiary structure (*right structure*). In addition, the conserved patch of amino acid sites overlapped with the binding site of binuclear Cu(II) for hydrolytic cleavage of the protein (8). The patch included the amino acid sites Gln91, Ser92, Ala94 and Thr95, all of which are cleaved by Cu(II) (8).

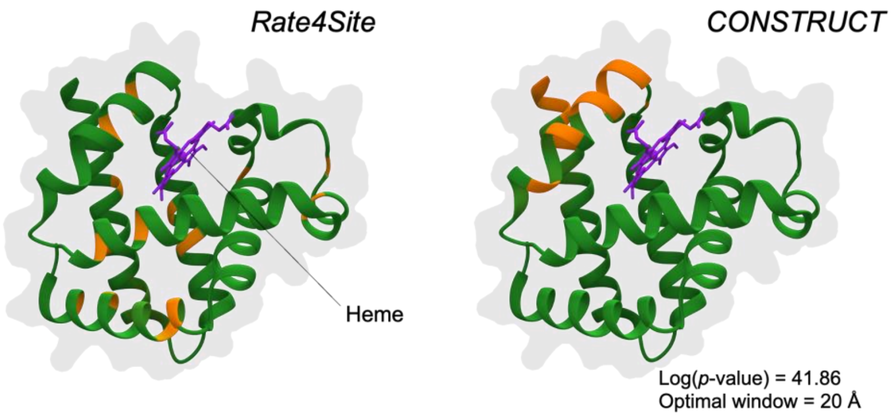

DHPS (Uniprot: Q25704, PDB ID: 6JWV)
The DHPS dataset consisted of 55 orthologous sequences. Rate4site revealed that the most conserved sites were largely distributed throughout the protein tertiary structure (*left structure*). However, these sites included Ser436 and Lys609, which are involved in pteroate and sulfa derivatives (9). CONSTRUCT was then applied, and the maximum strength of spatial correlation was detected at a distance of 19 Å, associated with a log(*p*-value) of 60.62. Again, the most conserved sites identified by CONSTRUCT formed a well-defined patch in the tertiary structure previously reported as the site of sulfa interaction (*right structure*). In particular, the patch included the amino acid sites Asn502, Asp539, Phe580, Lys609, Arg686 and His699, which are important for pteroate and/or sulfa derivative interaction (9).

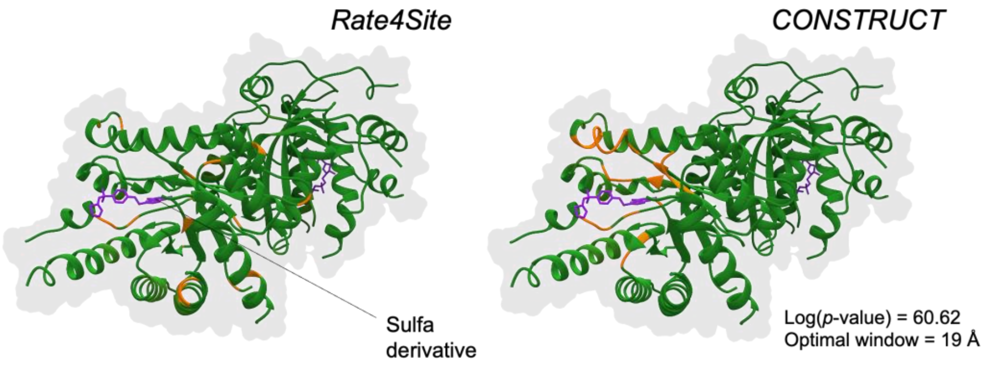

MAPK1 (Uniprot: P63086, PDB ID: 5UMO)
Rate4Site was first run on a dataset of 493 MAPK1 orthologous sequences. The most conserved amino acid sites were uniformly distributed throughout the tertiary structure (*left structure*). However, these conserved sites included Asp147, which is part of the catalytic loop and essential for ATP binding and catalysis (10). CONSTRUCT was then run on the same dataset. A patch of conserved amino acid sites was detected (log(*p*-value) = 50.73) at an optimal distance of 17 Å. This patch covered the catalytic site of the protein (*right structure*) (10). Especially, the patch included Arg146, Tyr185, Val186, Ala187, and Ser211, which are both phosphorylated sites and interacting sites with ATP (10).

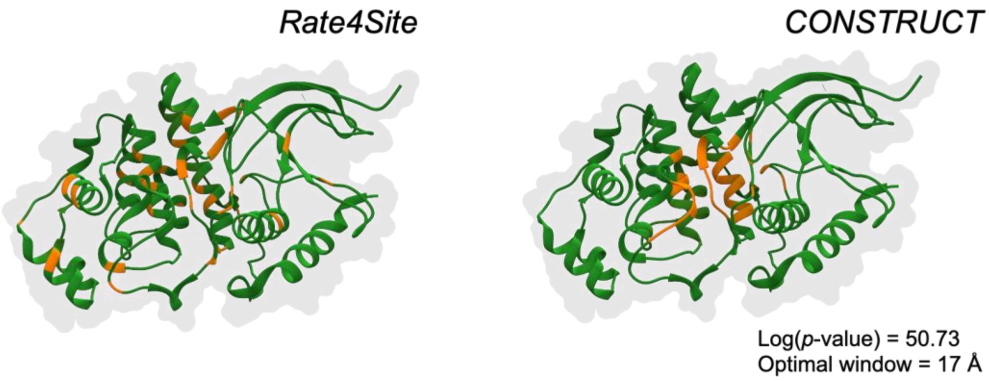

SGLT1 (Uniprot: P13866, PDB ID: 7SL8)
Rate4Site was first run on a dataset of 402 SGLT1 orthologous sequences. The most conserved amino acid sites were uniformly distributed throughout the tertiary structure (*left structure*). These conserved sites included Glu102 and Lys321, which are located in the glucose binding pocket (11). CONSTRUCT was then run on the same dataset. A patch of conserved amino acid sites was detected (log(*p*-value) = 88.76) at an optimal distance of 20 Å. This patch covered both the extracellular gate of the protein and the glucose binding pocket (*right structure*) (11). Especially, we found amino acid sites Asn78 and Tyr290, which have been shown to be involved in glucose binding; and Gly86, Leu452, Phe453, which are located at the extracellular gate of SGLT1 (11).

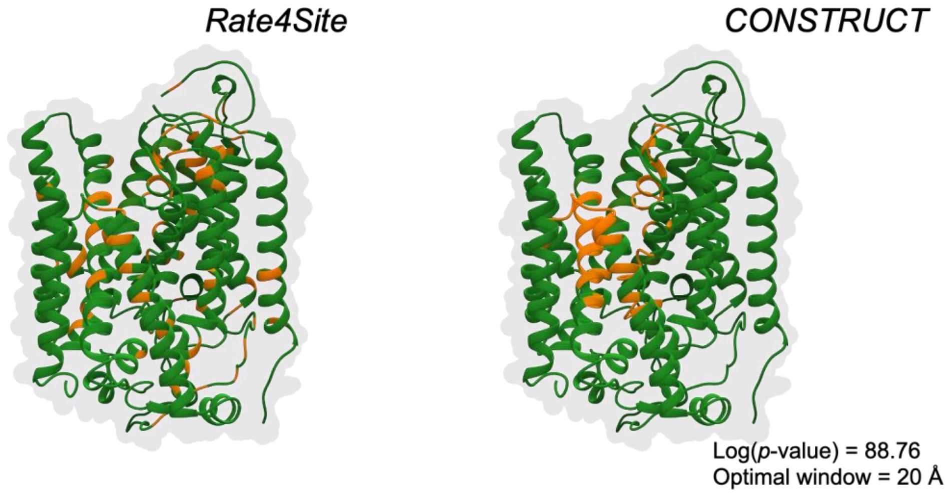

GTPase HRas (Uniprot: P01112, PDB ID: 5P21)

The HRas dataset consisted of 421 orthologous sequences. Rate4site revealed that the most conserved sites were largely distributed throughout the protein tertiary structure (*left structure*). However, these sites included Phe28, Asn116, Asp119, Thr144 and Ser145, which are involved in the interaction with GppNp molecule (12). CONSTRUCT was then applied, and the maximum strength of spatial correlation was detected at a distance of 17 Å, associated with a log(*p*-value) of 29.44. The most conserved sites identified by CONSTRUCT formed a well-defined patch in the tertiary structure previously reported as the interaction site between RAS and its effectors (*right structure*) (12). In particular, the patch included the amino acid sites Lys16, Ser17, Glu31, Tyr32, Asp33, Pro34, Thr35, Glu37, Asp38, Asp57, Gly60, Glu62 and Glu63, all of which were previously reported to be involved in such interactions (12).

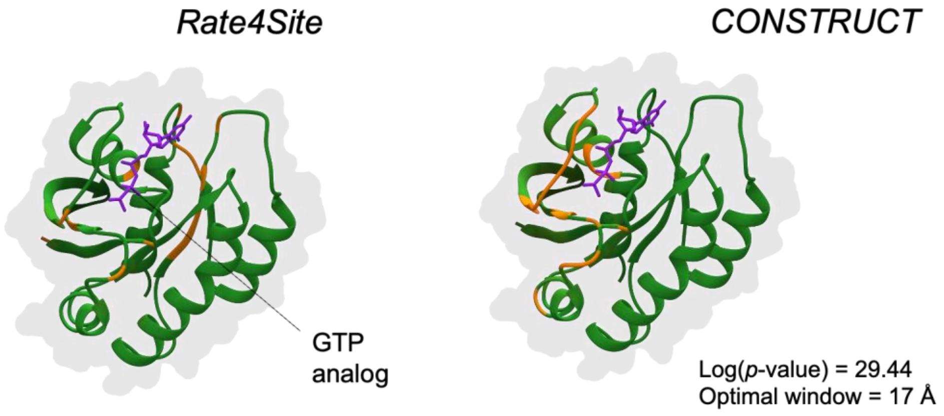

MDM2 (Uniprot: Q00987, PDB: 1YCR)

The MDM2 dataset consisted of 376 orthologous sequences. Using only the Rate4Site algorithm, conserved amino acid sites were distributed throughout the protein structure (*left structure*). Using CONSTRUCT, a spatial correlation of site-specific substitution rates was detected (best distance fixed at 10 Å, associated with a log(p-value) of 15.07). The most conserved sites identified by CONSTRUCT formed a well-defined patch in the tertiary structure, overlapping with the MDM2 cleft, which directly interacts with the p53 protein (13, 14). Therefore, the predicted conserved patch is likely to be important for the MDM2-p53 interaction.

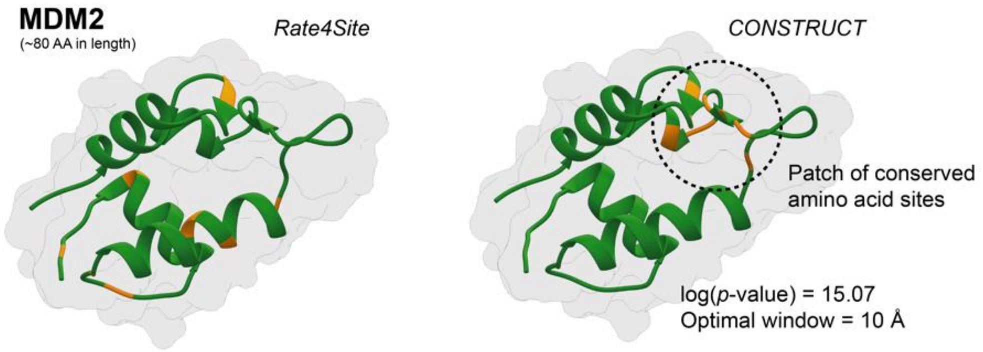

cAMP (Uniprot: P17612, PDB: 4WB5)

The cAMP dataset consisted of 254 orthologous sequences. With Rate4Site, the most conserved amino acid sites were uniformly distributed throughout the tertiary structure (*left structure*). Using CONSTRUCT, a patch of conserved amino acid sites was detected, maximized at a distance of 17 Å (log(p-value) = 78.87). The patch in the tertiary structure corresponds to interaction surface with ATP. In particular, the patch included the amino acid sites Arg165, Asp166, Lys168, Thr195, Tyr204, Leu205, Glu208, Tyr215, Asp220, Trp222, and Arg280, which are important for catalytic activity (15, 16).

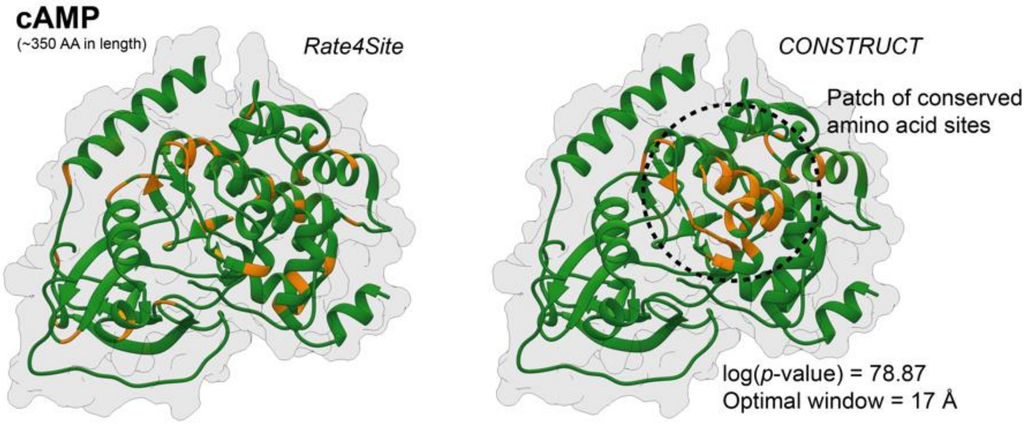

Torsin-1B (Uniprot: O14657, no PDB ID)

To run CONSTRUCT, the tertiary structure of the protein of interest is required. However, only a limited number of protein structures have been solved experimentally. To address this, artificial intelligence-based approaches, such as Google DeepMind's AlphaFold, have been developed to accurately predict protein tertiary structures. We wanted to see if CONSTRUCT could precisely identify functional regions based on predicted structures. We studied the protein Torsin-1B, a member of the AAA+ (ATPases Associated with diverse cellular Activities) superfamily, which is involved in several cellular processes, including proper protein folding, maintenance of cellular homeostasis, and possibly the secretory pathway (17). The predicted tertiary structure of Torsin-1B suggested a central domain responsible for ATP binding and hydrolysis, which drives the conformational changes required for its function (*panel A*). It has been reported that Glu178Gln results in a loss of ATPase activity while enhancing the interaction with TOR1AIP2 (17). The interaction with ATP was verified using CB-Dock2 (default parameters) (18), which predicted the interaction to occur within the protein cavity, involving Glu178 and 21 other amino acid sites, with an estimated binding affinity of -8.8 kcal/mol (*panel B*).

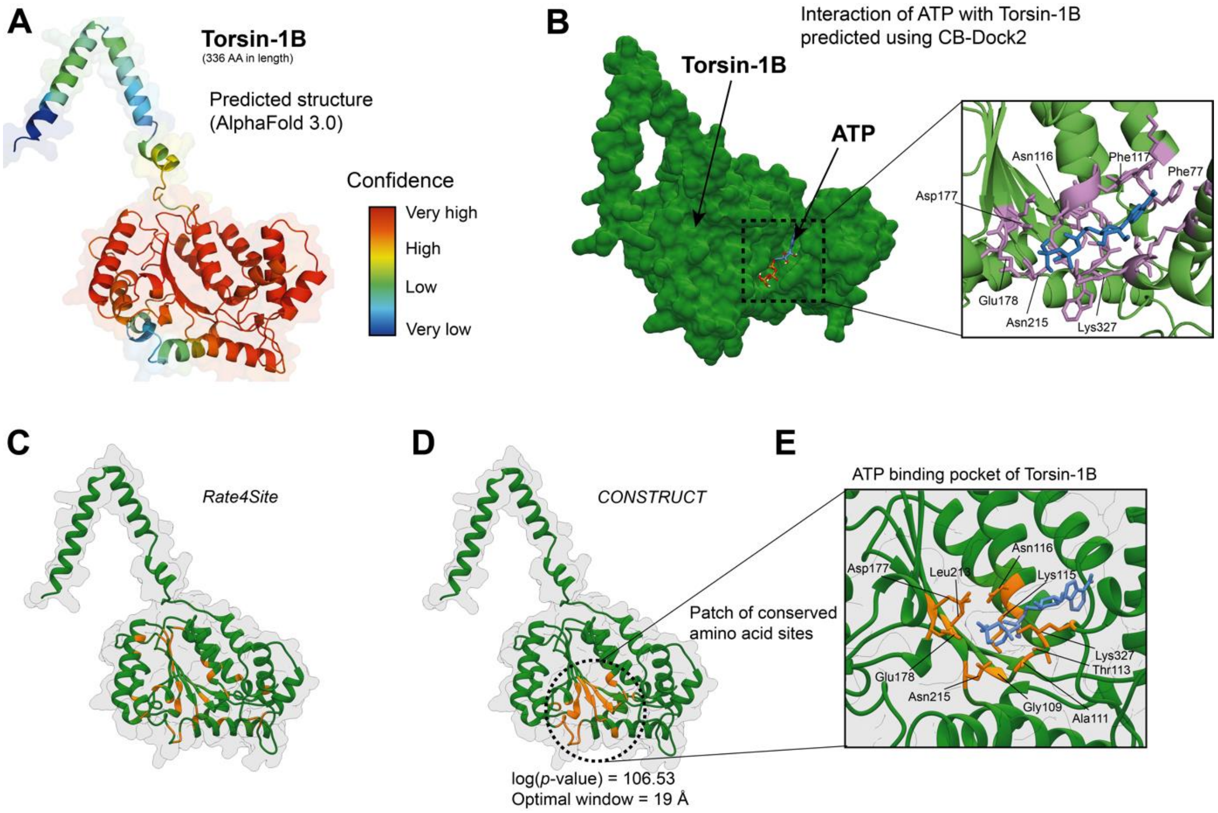

Identification of patch of conserved amino acid sites using a predicted tertiary structure. (A) Predicted tertiary structure of the Torsin-1B protein. The color scale corresponds to the confidence of the prediction for each amino acid site according to AlphaFold 3.0. **(B)** Predicted interaction of ATP with Torsin-1B. Torsin-1B and ATP molecule are shown as surface and stick, respectively. A zoom of the ATP binding pocket shows likely important amino acid sites of Torsin-1B involved in the interaction. **(C** and **D)** Location of the 10% most conserved amino acid sites (colored in orange) in the tertiary structure of Torsin-1B according to Rate4Site and CONSTRUCT, respectively. **(E)** Zoom on some amino acid sites in the predicted ATP-binding pocket of Torsin-1B, which belong to the patch of conserved amino acid sites detected by CONSTRUCT.

Rate4Site was first run on a dataset of 299 Torsin-1B orthologous sequences, revealing that the most conserved amino acid sites were uniformly distributed throughout the tertiary structure of Torsin-1B (*panel C*). CONSTRUCT was then run on the same dataset. A patch of conserved amino acid sites was detected (log(*p*-value) = 106.53) at an optimal distance of 19 Å. This patch of conserved amino acid sites covered the predicted ATP interaction surface of Torsin-1B (*panel D*). The patch included the amino acid sites Gly109, Ala111, Thr113, Lys115, Asn116, Asp177, Glu178, Leu213, Asn215, and Lys327, all of which are involved in the predicted ATP interaction (*panel E*). Notably, the patch contained Glu178 (ranked as the second most conserved site based on spatially correlated site-specific substitution rates), which is essential for the ATPase activity of Torsin-1B (17).

KEAP1 (Uniprot: Q14145, PDB: 2FLU)

Application of the Rate4Site algorithm revealed that conserved amino acid sites are uniformly distributed across the three-dimensional structures of the KEAP1 propeller, based on 135 orthologous sequences. CONSTRUCT identified a conserved patch at the shallow pocket on the surface of the propeller domain, which was experimentally shown to be the interaction surface with Nrf2 (optimal distance of 14 Å and a log(*p*-value) of 74.63). The patch included five amino acids reported to be essential for interaction with Nrf2: Ser363, Arg380, Asn382, Arg415 and Ser508 (19).

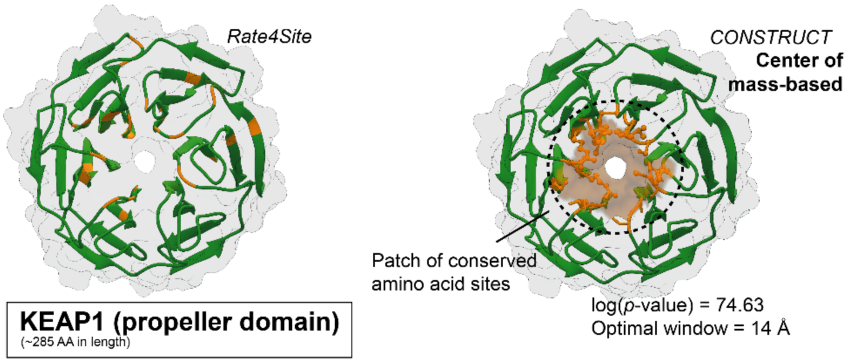

GDP-mannose transporter 1 (Uniprot: P40107, PDB: 5OGE)

Application of the Rate4Site algorithm revealed that conserved amino acid sites are uniformly distributed across the three-dimensional structures of GDP-mannose transporter 1, based on 366 orthologous sequences. With CONSTRUCT, a conserved amino acid patch was observed within the transporter channel (optimal distance of 8 Å and a log(*p*-value) of 54.76), indicating the interaction zone with the transporter's target molecule. The patch included five amino acids important for the interaction with GDP-mannose or GMP substrates were located within this patch: Lys118, Ser269, Tyr281, Gly285 and Lys289 (20).

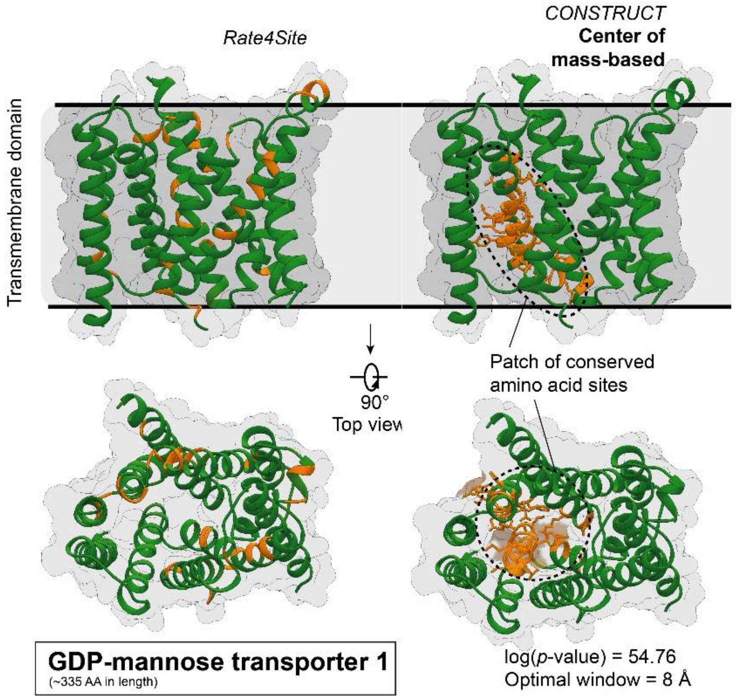

References

1. Brand,S.E., Scharlau,M., Geren,L., Hendrix,M., Parson,C., Elmendorf,T., Neel,E., Pianalto,K., Silva-Nash,J., Durham,B., *et al.* (2022) Accelerated Evolution of Cytochrome c in Higher Primates, and Regulation of the Reaction between Cytochrome c and Cytochrome Oxidase by Phosphorylation. *Cells*, **11**, 4014.
2. Bushnell,G.W., Louie,G.V. and Brayer,G.D. (1990) High-resolution three-dimensional structure of horse heart cytochrome c. *J Mol Biol*, **214**, 585–595.
3. Döpner,S., Hildebrandt,P., Rosell,F.I., Mauk,A.G., von Walter,M., Buse,G. and Soulimane,T. (1999) The structural and functional role of lysine residues in the binding domain of cytochrome c in the electron transfer to cytochrome c oxidase. *Eur J Biochem*, **261**, 379–391.
4. Moreno-Beltrán,B., Díaz-Moreno,I., González-Arzola,K., Guerra-Castellano,A., Velázquez-Campoy,A., De la Rosa,M.A. and Díaz-Quintana,A. (2015) Respiratory complexes III and IV can each bind two molecules of cytochrome c at low ionic strength. *FEBS Lett*, **589**, 476–483.
5. Yuthavong,Y. (2002) Basis for antifolate action and resistance in malaria. *Microbes and Infection*, **4**, 175–182.
6. Sirawaraporn,W., Sathitkul,T., Sirawaraporn,R., Yuthavong,Y. and Santi,D.V. (1997) Antifolate-resistant mutants of Plasmodium falciparum dihydrofolate reductase. *Proc Natl Acad Sci U S A*, **94**, 1124–1129.
7. Vanichtanankul,J., Taweechai,S., Yuvaniyama,J., Vilaivan,T., Chitnumsub,P., Kamchonwongpaisan,S. and Yuthavong,Y. (2011) Trypanosomal Dihydrofolate Reductase Reveals Natural Antifolate Resistance. *ACS Chem. Biol.*, **6**, 905–911.
8. Zhang,L., Mei,Y., Zhang,Y., Li,S., Sun,X. and Zhu,L. (2003) Regioselective cleavage of myoglobin with copper(II) compounds at neutral pH. *Inorg Chem*, **42**, 492–498.
9. Chitnumsub,P., Jaruwat,A., Talawanich,Y., Noytanom,K., Liwnaree,B., Poen,S. and Yuthavong,Y. (2020) The structure of Plasmodium falciparum hydroxymethyldihydropterin pyrophosphokinase-dihydropteroate synthase reveals the basis of sulfa resistance. *The FEBS Journal*, **287**, 3273–3297.
10. Zhang,F., Strand,A., Robbins,D., Cobb,M.H. and Goldsmith,E.J. (1994) Atomic structure of the MAP kinase ERK2 at 2.3 A resolution. *Nature*, **367**, 704–711.
11. Han,L., Qu,Q., Aydin,D., Panova,O., Robertson,M.J., Xu,Y., Dror,R.O., Skiniotis,G. and Feng,L. (2022) Structure and mechanism of the SGLT family of glucose transporters. *Nature*, **601**, 274–279.
12. Pai,E.F., Krengel,U., Petsko,G.A., Goody,R.S., Kabsch,W. and Wittinghofer,A. (1990) Refined crystal structure of the triphosphate conformation of H-ras p21 at 1.35 A resolution: implications for the mechanism of GTP hydrolysis. *The EMBO Journal*, **9**, 2351–2359.
13. Kussie,P.H., Gorina,S., Marechal,V., Elenbaas,B., Moreau,J., Levine,A.J. and Pavletich,N.P. (1996) Structure of the MDM2 oncoprotein bound to the p53 tumor suppressor transactivation domain. *Science*, **274**, 948–953.
14. Moll,U.M. and Petrenko,O. (2003) The MDM2-p53 interaction. *Mol Cancer Res*, **1**, 1001–1008.
15. Turnham,R.E. and Scott,J.D. (2016) Protein kinase A catalytic subunit isoform PRKACA; History, function and physiology. *Gene*, **577**, 101–108.
16. Yang,J., Ten Eyck,L.F., Xuong,N.H. and Taylor,S.S. (2004) Crystal structure of a cAMP-dependent protein kinase mutant at 1.26A: new insights into the catalytic mechanism. *J Mol Biol*, **336**, 473–487.
17. Rose,A.E., Zhao,C., Turner,E.M., Steyer,A.M. and Schlieker,C. (2014) Arresting a Torsin ATPase reshapes the endoplasmic reticulum. *J Biol Chem*, **289**, 552–564.
18. Liu,Y., Yang,X., Gan,J., Chen,S., Xiao,Z.-X. and Cao,Y. (2022) CB-Dock2: improved protein-ligand blind docking by integrating cavity detection, docking and homologous template fitting. *Nucleic Acids Res*, **50**, W159–W164.
19. Canning,P., Sorrell,F.J. and Bullock,A.N. (2015) Structural basis of Keap1 interactions with Nrf2. *Free Radic Biol Med*, **88**, 101–107.
20. Parker,J.L. and Newstead,S. (2017) Structural basis of nucleotide sugar transport across the Golgi membrane. *Nature*, **551**, 521–524.
