# Supplementary material for: CONSTRUCT: an algorithmic tool for identifying functional or structurally important regions in protein tertiary structure
Source: Bioinformatics. 2025 Apr 12;41(4):btaf166. doi: 10.1093/bioinformatics/btaf166 (PMC12034385; doi:10.1093/bioinformatics/btaf166)
Supplement: btaf166_Supplementary_Data [file btaf166_supplementary_data.zip › supplementary_tables.pdf]

Supplementary Table S1 – Analysis duration for each case study.

Protein	Number of orthologous sequences	Size of the alignment	Analysis duration (s)
Cytochrome c	853	103	423
MDM2	376	109	88
KEAP1 (propeller)	135	285	35
DHFR (N-terminal domain)	366	617	571
Myoglobin	454	153	180
cAMP-dependent protein kinase A	254	334	182
DHPS (catalytic domain)	55	641	24
CFTR	415	1,463	1,411
MAPK1	493	365	701
SGLT1	402	667	1,044
Torsin-1B	299	338	295
YddG	383	277	294
GDP-mannose transporter 1	366	322	310
GTPase Hras	421	166	202

Supplementary Table S2 – List of software and packages used in CONSTRUCT.

Software	Version	Link
Rate4Site	3.0.0	https://www.tau.ac.il/~itaymay/cp/rate4site.html
Python	3.10.12	https://www.python.org/
R	4.1.2	https://www.r-project.org/
**Python packages**		
Tkinter	8.6	https://docs.python.org/fr/3/library/tkinter.html
Customtkinter	0.3	https://pypi.org/project/customtkinter/0.3/
**R packages**		
Tidyverse	2.0.0	https://www.tidyverse.org/
Bio3d	2.4-4	http://thegrantlab.org/bio3d/
BiocManager	1.30.23	https://www.bioconductor.org/
Msa	1.26.0	https://bioconductor.org/packages/release/bioc/html/msa.html

Note – All the packages and software (except Python and R) can be easily installed using a bash script provided with CONSTRUCT.

Supplementary Table S3 – Results of CONSTRUCT on simulated cases with expected functional patch.

	Proportion (according to protein size)					
**Log(*p*-value)**	**50-100**	**100-200**	**200-300**	**300-400**	**400-500**	**Total**
**≥ 8**	91.5	97.0	99.0	98.5	99.0	98.0
**< 8**	8.5	3.0	1.0	1.5	1.0	2.0

Supplementary Table S4 – Results of CONSTRUCT on simulated cases after random permutations of site-specific substitution rates.

	Proportion (according to protein size)					
**Log(*p*-value)**	**50-100**	**100-200**	**200-300**	**300-400**	**400-500**	**Total**
**≥ 8**	3.74	1.37	0.64	0.44	0.33	1.30
**< 8**	96.26	98.63	99.36	99.56	99.67	98.70
